# Best serum biomarker combination for ovarian cancer classification

**DOI:** 10.1186/s12938-018-0581-6

**Published:** 2018-11-06

**Authors:** Hye-Jeong Song, Eun-Suk Yang, Jong-Dae Kim, Chan-Young Park, Min-Sun Kyung, Yu-Seop Kim

**Affiliations:** 1 Department of Convergence Software, Hallym University, Chuncheon, South Korea; 2 Bio-IT Research Center, Hallym University, Chuncheon, South Korea; 3 Department of Obstetrics and Hynecology, Hallym University Medical Center, Hwaseong, South Korea

**Keywords:** Marker selection, Classification, Ovarian cancer, Logistic regression, CA-125

## Abstract

**Background:**

Screening test using CA-125 is the most common test for detecting ovarian cancer. However, the level of CA-125 is diverse by variable condition other than ovarian cancer. It has led to misdiagnosis of ovarian cancer.

**Methods:**

In this paper, we explore the 16 serum biomarker for finding alternative biomarker combination to reduce misdiagnosis. For experiment, we use the serum samples that contain 101 cancer and 92 healthy samples. We perform two major tasks: Marker selection and Classification. For optimal marker selection, we use genetic algorithm, random forest, T-test and logistic regression. For classification, we compare linear discriminative analysis, K-nearest neighbor and logistic regression.

**Results:**

The final results show that the logistic regression gives high performance for both tasks, and HE4-ELISA, PDGF-AA, Prolactin, TTR is the best biomarker combination for detecting ovarian cancer.

**Conclusions:**

We find the combination which contains TTR and Prolactin gives high performance for cancer detection. Early detection of ovarian cancer can reduce high mortality rates. Finding a combination of multiple biomarkers for diagnostic tests with high sensitivity and specificity is very important.

## Background

Ovarian cancer is the eighth most common cancer and has the fifth fatality-to-case ratio in United States. It is also undetected until it goes at late stage. According to a statistics of Centers for Disease Control and Prevention (CDC) in 2012, about 20 thousands women in United States were diagnosed with ovarian cancer, and about 75% died from it. In addition, when ovarian cancer is found in its early stage, the probability of 5-year survival yields up to 92%. However the early detection rate is only 19%. It is clarify that the early detection of ovarian cancer improves the clinical output [1, 2].

For early diagnosis, many researches have been performed: finding multiple biomarkers [3], early detection using menopausal information [4], and finding optimal combination using machine learning algorithms [3, 5]. Specifically, many of them have been developed for distinguishing between benign and cancer [3–7].

One of the most popular screening test for ovarian cancer is CA-125 blood test. CA-125 is a protein in the blood. The level of CA-125 is high from many women with ovarian cancer. CA-125 is also useful for guiding treatment to patients with ovarian cancer, since a high level of CA-125 decrease if treatment is working well [8–10].

However, checking CA-125 level has led to misdiagnosis of ovarian cancer. The problem with using CA-125 for screening test for ovarian cancer is that common conditions other than cancer can also cause the level of CA-125 high. In addition, someone who has ovarian cancer does not have high level of CA-125.

For detecting ovarian cancer, in previous studies, we shows the multiple biomarker has high performance rather than single biomarker [3–7].

In this paper, the goal of our experiment is to find alternative biomarker combination which shows high diagnosis performance, using a variety of machine learning techniques instead of CA-125. We perform two major tasks. Each task describes as follows:

1. We search the optimal marker combinations from 16 serum biomarker. Using 4 different algorithms, we select the best combination from each combination set.
2. We compare the methods, which is widely used for classification, of optimal combination for distinguishing normal and cancer samples.

## Methods

Serum samples were obtained from 101 patients with ovarian cancer and from 92 healthy women provided through Hallym University Chuncheon Sacred Heart Hospital. To validate our approach, we do not care about cancer stage, and the state of menopause which is important factor associated with the risk of malignancy [11, 12]. The 16 serum biomarkers, which is commonly discussed for ovarian cancer researches, are used to our experiment [12–14].

To select optimal marker combination which can diagnose cancer and normal data, we use four algorithms: random forest (RF) [15], genetic algorithm (GA) [16], T-test and logistic regression (LR) [17]. The size of combination is set from 2 to 4 for reducing a time consuming. The top marker combinations for each algorithm are computed to fivefold cross-validation. We repeated it 1000 times in order to decrease the deviation of the result. The final best marker combinations are selected to average receiver operating characteristic (ROC) Area Under the Curve (AUC). ROC AUC is described on the next subsection in a detail.

With the selected optimal marker sets, for each combination size, we apply the three method, which is commonly used for classification: linear discriminant analysis (LDA) [18], K-nearest neighbor (KNN) [19] and logistic regression (LR). We compare the accuracy for classification between normal and cancer data.

### Receiver operating characteristic area under the curve (ROC AUC)

In order to assess the test performance, sensitivity and specificity is commonly used and through two indicators, we can find how well a classifier can distinguish between patients and healthy people. When a certain diagnosis system is used, sensitivity is a measure that how well the system distinguish the samples, which is associated with condition. Specificity is a measure that how well the system distinguish the samples, which does not have associated condition. In addition, ROC curve is widely used to determine the accuracy of diagnosis [20, 21].

**Fig. 1:**
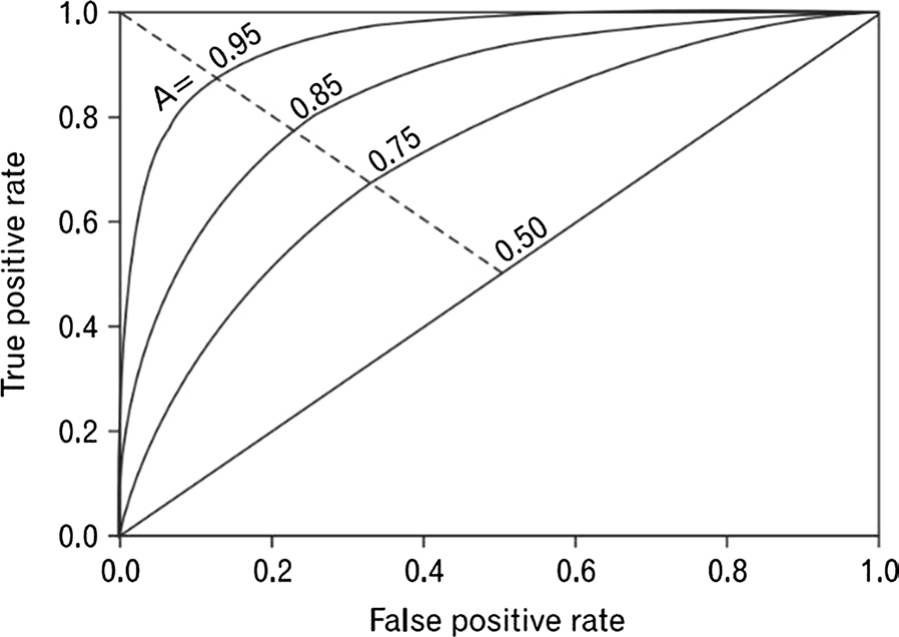
Receiver operating characteristic area under the curve (ROC AUC). The number on each curve indicates the measure of diagnosis accuracy

ROC is a plot that illustrates the performance of a binary classifier. In a plot, the x-axis indicates *1*-*specificity* and y-axis presents *sensitivity*. The accuracy of diagnosis is measured to AUC. Figure 1 shows the ROC graph for a settings of the decision criterion. According to AUC value, the quality of test is classified. The numbers on the curve present the degree of accuracy as follows; no discrimination (AUC < 0.5), fairly acceptable (0.5 < AUC < 0.7), excellent (0.7 < AUC < 0.9) and outstanding (0.9 < AUC).

## Results

In this section, we describe the result of each tasks: Marker selection and classification. For marker selection, we shows the performance of selected marker sets and analysis AUC values of combinations from single marker AUC values. For classification, we compare the three different classification methods.

### Marker selection results

Table 1 shows the optimal combination lists for the size of combination. The first column in Table 1 indicates algorithm for marker selection. Each describes as follows: GA is genetic algorithm, RF is random forest and LR is logistic regression. The second column presents the number of combinations. The listed combinations which ranges from 2 to 4, are selected to average AUC. Each combinations are chosen the highest AUC value from all possible combinations with the number of combination. The bold presents the highest AUC value in each algorithm.

**Table 1:** The AUC value for each combinations for each feature selection algorithm

Algorithm	Size	Combinations	AUC
GA	2	ApoCIII, TTR	0.86
	3	IL-6, CEA, OPN	0.90
	4	MIF, ApoAI, OPN, IL-6	*0.90*
RF	2	CA125, HE4-ELISA	0.92
	3	Prolactin, TTR, HE4-ELISA	*0.98*
	4	TTR, Prolactin, CA125, HE4-ELISA	0.98
T-Test	2	TTR, ApoCIII	0.95
	3	TTR, ApoCIII, Prolactin	0.98
	4	TTR, ApoCIII, Prolactin, OPN	*0.99*
LR	2	Prolactin, TTR	0.98
	3	ApoCIII, HE4-ELISA, Prolactin	0.99
	4	HE4-ELISA, PDGF-AA, Prolactin, TTR	*0.99*

The GA and RF yields 0.9 AUC value and 0.98 AUC value. With slight improvement, T-Test and LR perform 0.99 AUC value. In four results, GA have the lowest AUC value of 0.86 on 2 combination. The lowest performance among high score in each algorithm also perform in GA.

Except RF, the rest of algorithm shows the best AUC value to 4 combinations. However, there are no major differentiation of AUC value between 3 and 4 marker combinations. Intuitively, we find that it is not necessary to use 4 marker combination.

**Fig. 2:**
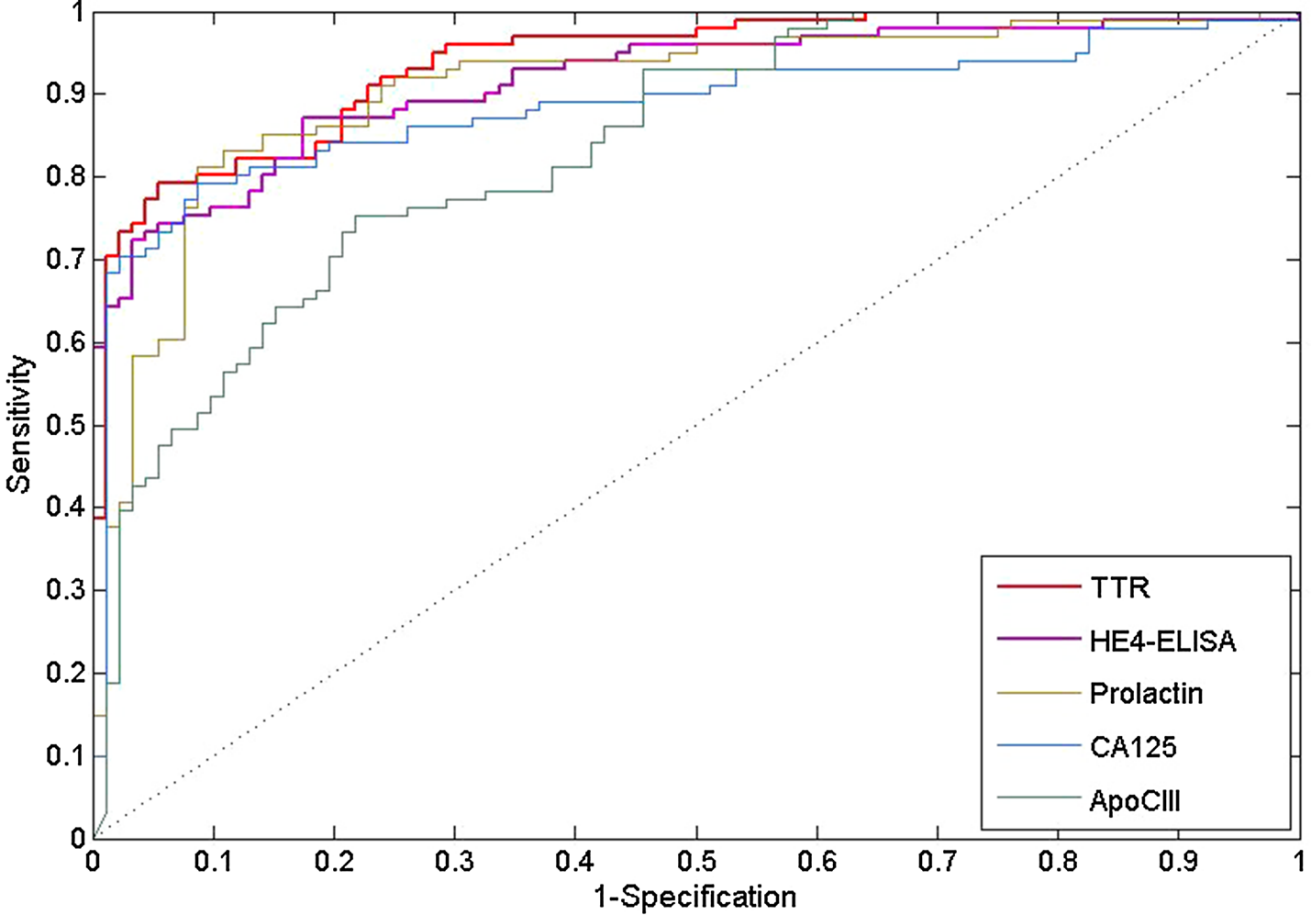
ROC curve for the best five single marker

Table 2 describes top 10 single markers sorted to AUC value. TTR, HE4-ELISA and Prolactin which have AUC value bigger than 0.9, are well captured to important biomarker when selecting the marker combination, except GA. We analysis that GA shows the low AUC compared to the rest of combinations, since combined markers have AUC value which is even less than 0.8. Figure 2 shows ROC curve for the best 5 single marker.

**Table 2:** Top 10 AUC value of single marker computed to logistic regression

Marker	AUC
TTR	0.94
HE4-ELISA	0.92
Prolactin	0.91
CA125	0.88
ApoCIII	0.84
MIF	0.80
OPN	0.78
PDGF-AA	0.76
IL-6	0.73
CRP	0.71

### Classification

Table 3 shows the accuracy of classifications for each marker combination. In marker sets selected by GA, 2 combination shows the best performance of 0.88 using KNN. The 3 combination performs the lowest accuracy of 0.81 in overall results. In marker sets chosen by RF, 3 and 4 combination yield almost same accuracy using LR. The high score of 2 combination and 3, 4 combination display a significant gap with 0.12. Marker sets chosen by T-Test also shows totally same performance of 0.95 using LR. In optimal marker combinations using LR, a classifier using LR yields 0.95 accuracy, which is same as T-Test. However, a classifier using logistic regression shows the stable performance across all combination size.

**Table 3:** Accuracy of three classification method for each marker combination

Algorithm	Combinations	LDA	KNN	LR
GA	ApoCIII, TTR	0.88	*0.88*	0.87
	IL-6, CEA, OPN	0.67	0.79	*0.81*
	MIF, APoAI, OPN, IL-6	0.74	*0.86*	0.83
RF	CA125, HE4-ELISA	0.69	*0.83*	0.78
	Prolactin, TTR, HE4-ELISA	0.91	0.93	*0.95*
	TTR, Prolactin, CA125, HE4-ELISA	0.92	0.91	*0.94*
T-Test	TTR, ApoCIII	0.88	*0.88*	0.87
	TTR, ApoCIII, Prolactin	0.93	0.93	*0.95*
	TTR, ApoCIII, Prolactin, OPN	0.93	0.93	*0.95*
LR	Prolactin, TTR	0.91	0.92	*0.93*
	ApoCIII, HE4-ELISA, Prolactin	0.93	*0.94*	0.93
	HE4-ELISA, PDGF-AA, Prolactin, TTR	0.92	0.94	*0.95*

Not surprisingly, the GA algorithm which has a lowest AUC value for marker selection, performs the lowest accuracy of 0.80 for 3 combination. All marker selection algorithm except GA, shows better performance for the 3 and 4 combinations rather than 2 combinations. The performance between 2 combination and 4 combination for RF, T-Test and LR are about 0.11, 0.6 and 0.2 apart, respectively. The classifier using logistic regression shows the outstanding performance in over 70% of marker sets. We also find that TTR and Prolactin contains in combination which shows the good performance.

## Discussion

In this paper, we present the exploration for the marker selection and classification between cancer and normal samples, using machine learning algorithms. For marker selection, we find all methods except genetic algorithm, can capture in combining marker sets a marker, which has a high AUC value. Among them, logistic regression shows high performance for all combinations in general. For classification, logistic regression also presents the highest accuracy. Logistic regression also shows the stable accuracy on classification. It indicates that logistic regression can capture optimal combination and classify two difference class well. The experimental results shows that logistic regression is an outstanding algorithm for both problem.

## Conclusions

We find the combination which contains TTR and Prolactin gives high performance for cancer detection. With the stability and accuracy, we can find Her-ELISA, PDGF-AA, Prolactin and TTR is the best biomarkers for classifying cancer samples from healthy to cancer data. Early detection of ovarian cancer can reduce high mortality rates. Finding a combination of multiple biomarkers for diagnostic tests with high sensitivity and specificity is very important. For future works, we can apply our approach to urine samples or can be considerer to highly influential factor for detecting ovarian cancer, such as age, the stage of cancer and the state of menopause.

## Abbreviations

RF: random forest
GA: genetic algorithm
ROC: receiver operating characteristic
AUC: area under the curve
LDA: linear discriminant analysis
KNN: K-nearest neighbor
LR: logistic regression
